# High Resected Gastric Volume and poorly controlled DM2 in laparoscopic sleeve gastrectomy

**DOI:** 10.1016/j.amsu.2018.10.034

**Published:** 2018-11-03

**Authors:** Federico Sista, Valentina Abruzzese, Stefano Guadagni, Sergio Carandina, Marco Clementi

**Affiliations:** a Dipartimento DISCAB, University of L'Aquila – San Salvatore Hospital, L'Aquila, Italy; b ELSAN, Surgical Obesity Center (CCO), Clinique Saint Michel, Toulon, France; c Dipartimento MESVA, University of L'Aquila – San Salvatore Hospital, L'Aquila, Italy

**Keywords:** Obesity, Sleeve gastrectomy, Volume resected, Bariatric surgery, Diabetes mellitus type 2, Hb1Ac

## Abstract

**Background:**

Our aim is to evaluate the effects of High Resected Gastric Volume(HRGV) on poorly Type 2 Diabetes Mellitus(DM2) after Laparoscopic Sleeve Gastrectomy(LSG).

**Methods:**

256 patients were divided into two groups according to the RGV: < 1500 mL(Group A: 131 pts) and > 1500 mL(Group B: 147 pts). % excess body mass index loss (%EBMIL), Fasting Blood Glucose (FBG), HbA1c, C peptide were assessed before surgery and at the 3rd day, 6th,12th,24th,36th month after LSG.

**Results:**

A significant difference in %EBMIL between the two groups at 24 and 36 months was found. RGV was not significantly associated with DM2 in the multivariate logistic regression. FBG levels showed no differences between the two groups. A significant decrease of Hb1Ac at 6 and 12 months was found in group B. The C-peptide level showed a significant reduction at 6 and 12 months in group B.

**Conclusion:**

The HRGV may play a role in the regulation of the glucose metabolism in the first year after LSG without influence in poorly DM2 control. Further studies are needed to confirm these findings.

## Highlights

• The study showed that the High Resected Gastric Volume (HRGV) play a role in the regulation of the glucose metabolism in the first year after LSG without influence in poorly DM2 control.

## 1 Introduction

Among all bariatric surgical procedures, the Laparoscopic Sleeve Gastrectomy (LSG) is one of the most effective for the long-lasting treatment of severe obesity and its related conditions [1]. Therefore, the effect of the LSG on DM2 has been documented in several studies [1,2]. Nevertheless, the glycemic control often occurs before the achievement of a significant weight loss, which suggests that the control of the glycemic status may be a direct effect of surgery rather than a secondary effect of the weight loss [2]. In fact, LSG appears to induce a significant hormonal change in glucose homeostasis by the removal of a large portion of the stomach [2,3].

Recently, some authors adopted age, body mass index (BMI), C-peptide level and duration of DM2 as predicting factors for the glycemic control after LSG [3], but currently there are some Authors [[4], [5], [6]] in literature reports that compare the resected gastric volume (RGV) with DM2 control with different results.

Recently, several studies documented a relationship between a RGV greater than 1200 ml, weight loss and comorbidities resolution after LSG compared to volume lower than 1200 ml [5,6]. However, no study shows if this correlation also exists for high-volume gastric resection (HGVR).

The aim of this prospective observational study was to establish a correlation between HRGV and DM2 control in a cohort of 256 patients during the first 3 years after surgery.

## 2 Materials and methods

### 2.1 Study design

This is a prospective observational study including morbidly obese patients who underwent LSG from April 2012 to January 2015 at Surgical obesity Center (Clinique Saint Michel, Toulon, France).

According to the National Institute of Health, all patients with a body mass index (BMI) ≥ 35 kg/m^2^ with at least 1-coexisting obesity-related comorbidity, were eligible for the study.

Other inclusion criteria were the following: diabetes duration less than 10 years since evidence in the literature suggests that DM lasting for more than 10 years is a negative prognostic factor for LSG effects on diabetes [6]; age between 20 and 60 years old; no immunosuppressive therapy; a poorly controlled DM2 defined by a glycated hemoglobin A1c (HbA1c) levels ≥7% and Fasting Blood Glucose (FBG) > 100 mg/dL after administration of hypoglycemic (oral and insuline) drugs for 6 months despite proper nutrition [7,8].

Patients were divided into two different groups, according to the RGV measured at the end of surgery. In Group A were enrolled patients with a RGV between 1100 ml and 1500 ml, in Group B patients with a RGV greater than 1500 ml.

**Fig. 1:**
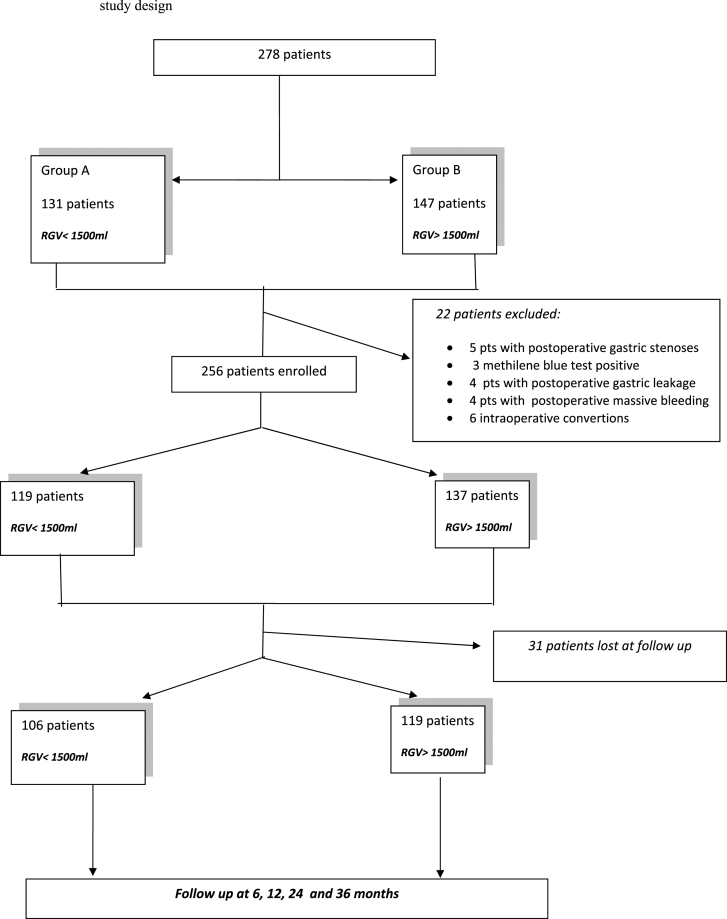
Study design.

For this reason, 278 patients meeting the inclusion criteria were enrolled but finally 225 patients completed the three-years follow-up and were assessed (Fig. 1).

The ethical committee of the Surgical Department of Clinique Saint Michel (Toulon), approved the study's protocol. All patients signed an informed consent form. According to the World Medical Association's Declaration of Helsinki 2013, the trial registration number is *researchregistry4289* (http://www.researchregistry.com).

Our Study meets the STROCSS criteria according to Agha RA et al. [9].

### 2.2 Surgical procedure and postoperative management

All procedures were performed laparoscopically using a four-port technique. Sleeve calibration was obtained by passing a 36–Fr gastric bougie and the stomach was transected with sequential firings of linear green and blue GIA reloads (Echelon^®^60 mm Ethicon Endo-Surgery). Resected stomach was extracted intact from the abdomen in a plastic bag, by enlarging the right subcostal incision.

In order to measure the RGV, a 16-Fr Foley catheter was inserted in the gastric antrum and a saline solution mixed with methylene blue was manually injected using a 50-ml syringe until leakage was detected on the staple line. A double suture was used to close the hole around the catheter to avoid liquid loosing. A simple manometric glass tube was used to evaluate the leak pressure in each specimen. The RGV was recorded when the staple line leakage occurred. All patients were double checked with the methylene blue test and an upper gastrointestinal transit on the 2nd postoperative day. If no leakage was detected, a liquid diet was started. The patients were discharged on the 5th postoperative day after eating mashed foods.

### 2.3 Follow up and end points

The postoperative follow up was conducted by a bariatric surgeon of our team at 1,3,6,12 and 24 months after the operation and once par year thereafter. The end points evaluated were excess body mass index loss (%EBMIL) and DM2.

The percentage of excess body mass index loss (%EBMIL) was calculated as follows: %EBMIL=(baseline BMI − follow up BMI)/(baseline BMI−ideal body weight). The Ideal Body Weight(IBW) of 25 kg/m^2^ was chosen, as proposed and accepted by several studies in literature [8], to describe the weight ranges associated with the maximum life expectancy.

Blood samples were collected from all patients before operation and 6, 12, 24 and 36 months after surgery. The laboratory tests included Fasting Blood Glucose(FBG), HbA1c and C-peptide. The diabetologists of our team monitored the patients every 3 weeks. A FBG<100 mg/dL and HbA1c level<6.5% without hypoglycemic drugs were considered as resolution of diabetes [10].

### 2.4 Statistical analysis

The study was designed as a prospective, observational, parallel group trial to compare two different clinical entities. Data were analyzed for normality of distribution with Shapiro-Wilk test. Since data distribution was not normal, mean values of overall differences were compared among the groups, by a non-parametric analysis of variance, Kruskal-Wallis test, and post-hoc analysis for comparisons of pairs of mean values with Mann-Whitney test with Bonferroni adjustment for multiple comparisons, and thus significance for the univariate analyses was assessed at p < 0.0167. Categorical variables, expressed as percentage, were compared by Chi square and *t*-test. Comparisons between groups were analyzed on an intention-to-treat basis.

Linear and logistic regression modeling were performed to determine the association between RGV and DM2 resolution at 36 months post-surgery, respectively. All regression models were adjusted for gender, age, initial obesity level (BMI) and RGV.

For all other analysis to the exclusion of post-hoc analysis a 5% significance level was adopted and the data analyzed using the Stata/IC12.1 statistical package.

## 3 Results

Patients’ demographic characteristics are showed in Table 1. The mean RGV was 931 ± 173 ml and 1489 ± 254 ml in Group A and Group B respectively.

**Table 1:** Characteristic of the patients.

	Groups			P value
	*Group A* (RGV 1100–1500 ml) n:106	*Group B* (RGV>1500 ml) n:119	*Total* n:225	
Age, mean ± SD	38.9±12.1	41.7±12.8	40.46±12.6	p = 0.92
BMI, mean ± SD	43.3 ± 7.8	47.2 ± 5.4	45.33 ± 4.7	p = 0.78
Pressure leak (cmH_2_O)	27.5 ± .8.2	26.1 ± 12.8	26.7 ± 14.3	P = 0.85
Sex, n(%) Female	70 (66)	81 (68.1)	151 (67.1)	p = 0.73
Duration of diabetes (month)	52 ± 11	49 ± 14	51 ± 15	p = 0.74
HbAc1% ± SD	8.3 ± 1.3	8.7 ± 1.4	8.5 ± 1.3	p = 0.41
FBG ±SD (mg/dl)	188 ± 43	191 ± 34	190 ± 37	p = 0.48
C-Peptide±SD (ng/ml)	2.4 ± 1	2.2 ± 0.9	2.3 ± 1	p = 0.11
Hypoglycemic drugs Use (%)	94 (88.7)	97 (81.6)	191 (84.9)	p = 0.57
Insuline Use (%)	50 (47.2)	62 (52.1)	112 (49.8)	p = 0.48

BMI: body mass index.

RGV: resected gastric volume.

SD: standard deviation.

HbAc1: glycated hemoglobin A1c.

FBG: Fasting Blood Glucose.

p > 0.05 no statistically significant difference between each group.

The distribution of RGV is shown in Table 2a: the analysis of variance didn't show a significant association between preoperative BMI and resected stomach volume and weight (p > 0.05).

**Table 2a:** The distribution of RGV.

BMI range	35–40	40–45	45–50	>50
Group A (RGV <1.500) n:106	13 (12.4%)	34(32.3%)	38(36.1%)	21(19.2%)
Group B (RGV >1.500) n:119	20 (16.8%)	33 (27.8%)	46(38.6%)	20 (16.8%)
Overall N:225	33 (14.7%)	67 (29.8%)	84(37.3%)	41 (18.2%)
P value	p=0.09	p=0.07	p=0.18	p=0.12

RGV was not correlated to preoperative BMI and biochemical parameter of insulin resistance: the values of HbA1c (8.3% vs. 8.7%, P = 0.41), FBG (188 vs. 191 mg/dL, P = 0.82), and C-peptide (2.5 vs. 2.2 ng/ml, P = 0.07) in both groups were similar (Table 1). Diabetes treatment was similar between the groups: 47.2% and 52.1% for Insulin and 88.7% and 81.6% for hypoglycaemic drugs respectively.

**Fig. 2:**
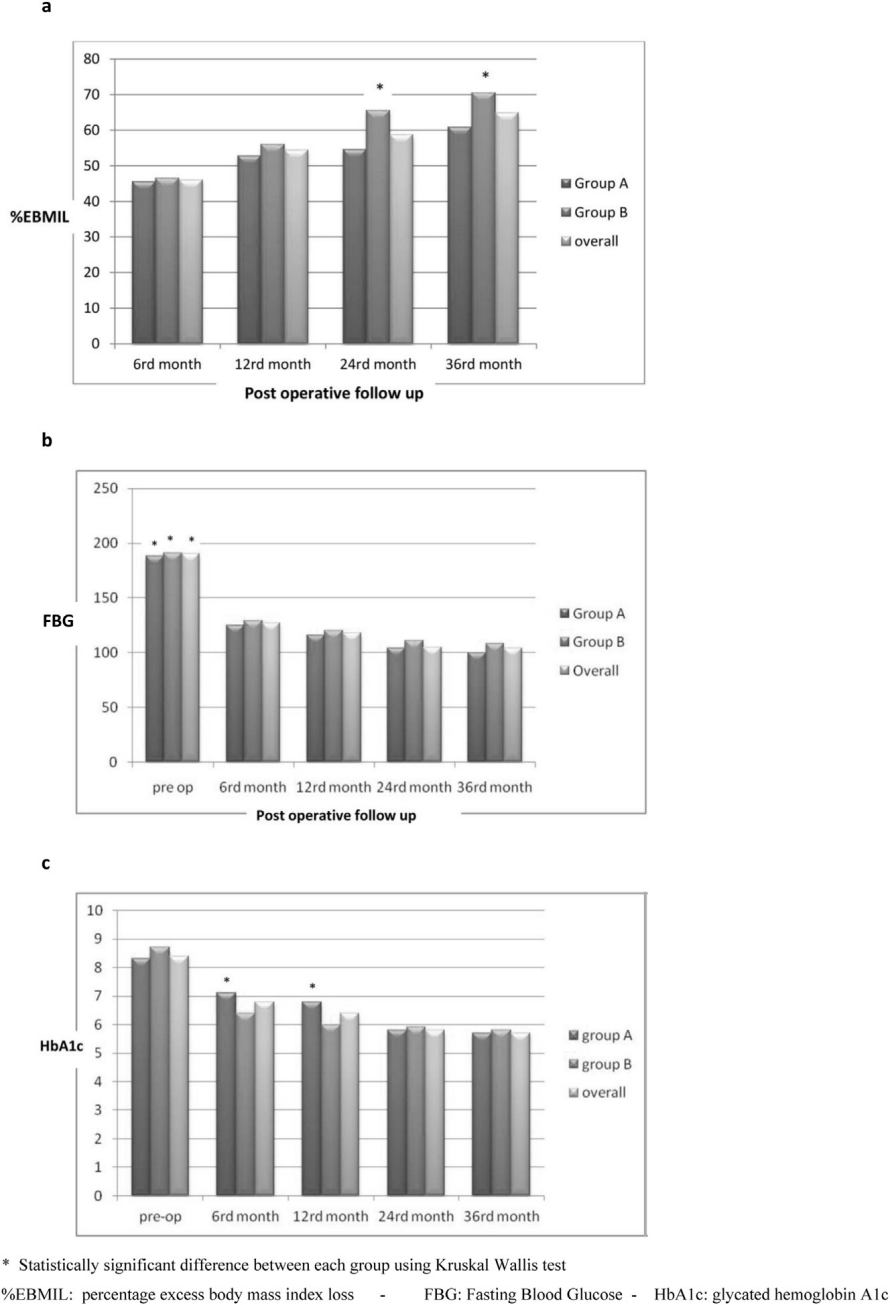
**a**: %EBMIL changes in the follow. **b**: FBG changes in the follow-up. **c**: HbA1c changes in the follow up. * Statistically significant difference between each group using Kruskal Wallis test. %EBMIL: percentage excess body mass index loss - FBG: Fasting Blood Glucose - HbA1c: glycated hemoglobin A1c.

Overall %EBMIL at 6, 12, 24 and 36 months was 46%, 54.5%, 58.7%, 64.9%, respectively. A statistically significant difference at 24 and 36 months, p = 0.03 and p = 0.02 respectively, was found (Fig. 2a).

The prevalence of the patients experimenting a resolution of DM2 at 6, 12, 24 and 36 months was 52.8%, 59.4%, 68.8%, 83% for group A and 48.7%, 57.1%, 73.1%, 79.8% for group B, with an overall resolution of 81.3% at 36 months (Table 2b).

**Table 2b:** Resolution of DM2.

Follow-up period	Groups			P value
	*Group A* (RGV< 1500 ml) n:106 n. pts (%)	*Group B* (RGV>1500 ml) n:119 n. pts (%)	*Total* n:225 n. pts (%)	
6 months	56 (52.8)	58 (48.7)	114 (50.7)	P=0.83
12 months	63 (59.4)	68 (57.1)	131 (58.2)	P=0.69
24 months	73 (68.8)	87 (73.1)	160 (71.1)	P=0.76
36 months	88 (83)	95 (79.8)	183 (81.3)	P=0.65

RGV: resected gastric volume.

p > 0.05 no statistically significant difference between each group.

No statistically significant differences in the proportion of patients with DM2 resolution were observed between the two groups during the follow up period. The results from the multivariate logistic regression modeling of DM2 resolution, controlled for the effects of sex, age, initial BMI and RGV are shown in Table 3. RGV was not significantly associated with DM2 resolution (95% CI = 3.78; 5.01; p = 0.04).

**Table 3:** Adjusted logistic regression of resolution of DM2 in the 36 months follow-up.

Age	O.R.	95% C.I.	P value
Sex	1.21	0.25; 6.31	p = 0.83
Age	1.05	0.97; 1.17	P = 0.06
Initial BMI	1.16	0.88; 1.32	p = 0.12
RGV	4.63	3.78; 5.01	p = 0.06
Hypoglycemic Drugs use	1.26	0.45; 2.31	p = 0.59
Insuline Use	1.57	0.48; 1.36	P = 0.23

BMI: body mass index.

RGV: resected gastric volume.

p > 0.05 no statistically significant difference between each group.

FBG levels were comparable for the two groups at all follow up points (Fig. 2b): In both groups, FBG levels were significantly decreased after six months, and the improvements were maintained through the 36-month evaluation.

A significant decrease of %HbA1c level was showed during the first year of follow up (Fig. 2c). Therefore, a statistically significant difference was observed between the two groups only at 6 and 12 months in favour of group B. The results obtained in group B at 6 months of follow up were similar to those obtained in group A at 12 months: 7.1% and 6.9% (Fig. 2c).

**Fig. 3:**
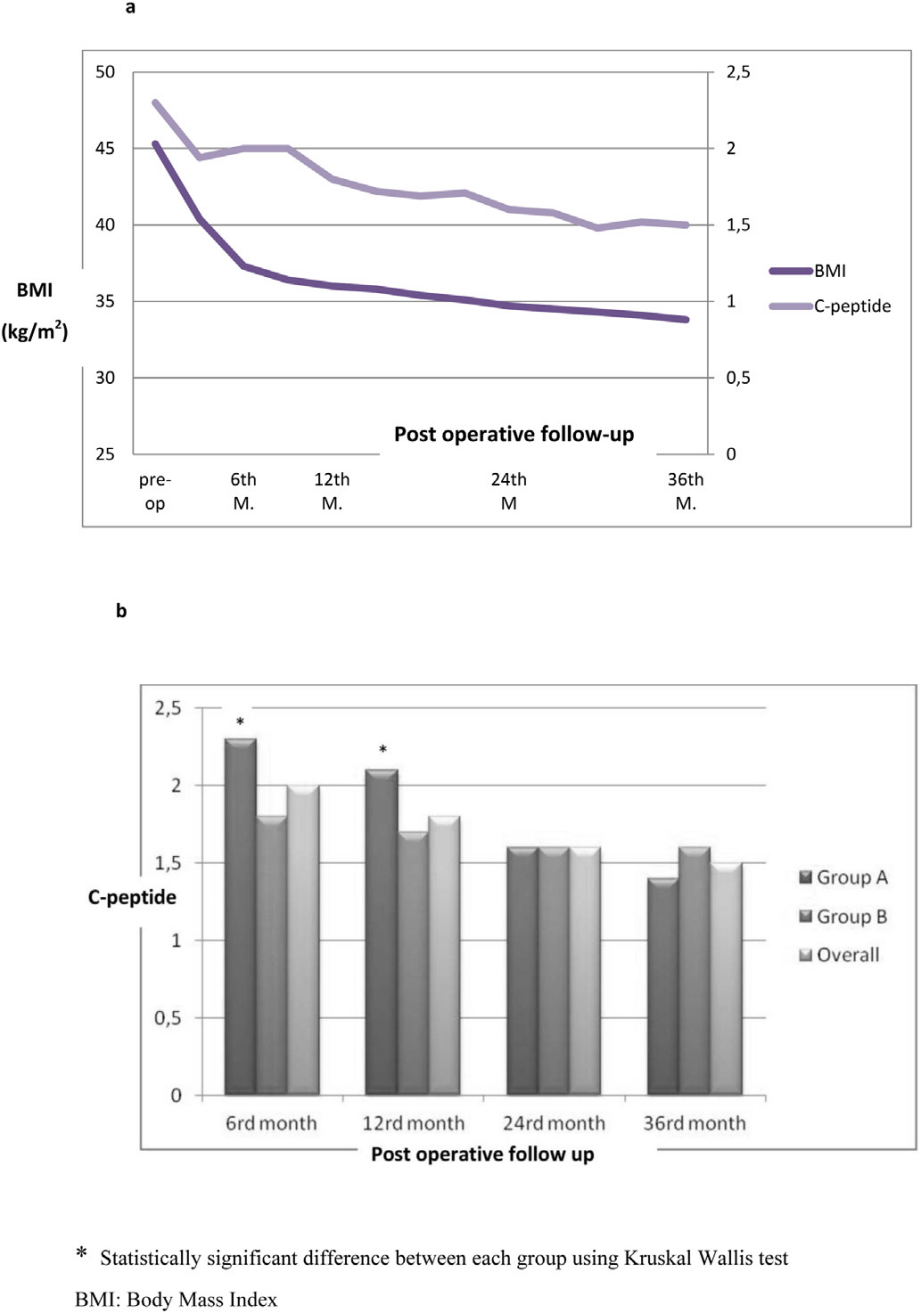
**a**: Change in BMI (kg/m2) and C-peptide (ng/ml) after LSG. **b**: C-peptide changes in the follow-up. * Statistically significant difference between each group using Kruskal Wallis test. BMI: Body Mass Index.

The C-peptide level was not correlated with the BMI (Fig. 3a). The mean preoperative C-peptide level was 2.3 ± 1 without any significant difference between the two groups: 2.4 ± 1 and 2.2 ± 0.9 respectively (Table 1), with a significant reduction only at 6 and 12 months of follow up (Fig. 3b).

## 4 Discussion

The Literature data reported that LSG was able to induce a remission of diabetes in 65–80% of patients, depending on patient population and length of follow-up [10,11]. In our multivariate logistic regression modeling of DM2 resolution we found that gender, age and initial BMI, did not play a role in diabetic remission (Table 3). In order to measure RGV, we manually injected saline solution in a 16-Fr Foley catheter inserted in the gastric antrum, until we had a leakage of the staple line. We had no difference between the leak pressure in the two groups and the gastric fundus was the most frequent site of leakage. Causey MW et al. [12] reported similar results about the leak pressure generated by the saline solution injection, suggesting the accuracy of this method of measurement with no significant prevalence in the location of the leak. Similar results were obtained by other authors [4,12,13].

The remaining gastric volume was not measured. In order to keep the sleeve volume constant and to decrease the relevance of this bias, surgical procedures were completed by the same surgeon using a standardized surgical technique. The stomach was always sectioned over a 36-Fr bougie tube and the resection was started 5 cm from the pylorus, trying to keep the sleeve volume constant [14].

There are some evidences in literature about the influence of RGV during LSG and its influence on weight loss and comorbidity resolution after surgery.

Significant differences in the results of LSG for RGV>1200 ml were showed in the literature [4,5,13]. Other authors have also shown that very high or low RGV cut-off do not determine significant differences between the samples [13,15]. Weiner and al. reported that a volume of the resected stomach inferior to 500 ml seemed to predict failure in weight loss or an early weight recovery [16].

In our study we used a RGV of 1500 ml as a cut-off to compare the results of LSG: we have chosen experimentally a cut off 1500 ml with a minimum RGV of 1100 ml for Group A because several studies [4,13] show that the average range of RGV is between 900 ml and 1400 ml with a median of Gaussian distribution around 1100 ml and other authors have described HRGV values above 1500–1800 ml [13,15]. The Authors believe that, although arbitrary, an RGV>1500 defines well a High Gastric Resection Volume.

The positive correlation between RGV and weight loss is still debated [4,5,13,14,16]. Some authors confirmed the positive relationship between RGV and weight loss [5,6,13], while in others this correlation was not found [4,17]. In the present study, we recorded a significant positive correlation between RGV and %EBMIL, which started one year after surgery and continued at 24 and 36 months of follow-up in patients with a higher volume of gastric resection. This evidence is also confirmed by the absence of correlation between the initial BMI and the RGV (Table 2a). These data showed that higher BMI does not correlate with larger volume of resections. In other words, patients with larger BMIs don't have larger volume stomachs. The authors believe that this evidence is very important because it reinforces the idea of metabolic surgery and that obesity is very complex, multifactorial disease.

The control of the glycemic status may be a direct effect of metabolic surgery rather than a secondary effect of weight loss [16,18]. Different hypothesis have been made concerning neuro-humoral changes related to gastric resection after LSG [[17], [18], [19]].

Several studies suggested that the changes on Ghrelin, Glucagon Like Peptide-1 (GLP-1) and Peptide YY (PYY) may be the major mechanisms of the antidiabetic effect after LSG [8,9,19].

This would suggest that a bigger RGV determines a greater depletion of ghrelin-secreting cells, thus inducing a higher reduction in ghrelin plasma levels. Therefore, a resection of larger gastric volumes could lead to more significant hormonal changes, thus improving insulin release and decreasing insulin peripheral resistance. Some evidences in Literature [6] showed these data concluding that RGV > 1200 ml had a better results in term of DM2 resolution.

However, in the present study we did not record a statistically significant difference between the two groups in the amount of patients who experienced DM2 resolution. We believe that a reason may be there are not difference in term of hormonal change for a cut off of 1500 ml. In other word for HRGV the gastric resection does not influence the secretion of gastrointestinal hormones and his the antidiabetic effect after LSG. Similarly, Sing and al [13]. showed in a prospective study an overall DM2 resolution in 82.9% of patients, without differences for RGV with a cut off of 1700 ml.

A significant reduction in C-peptide levels was recorded in group B during the first 12 months of follow-up, but this difference was not confirmed at 24 and 36 months. Since HbA1c and C-peptide are respectively expressions of insulin-resistance and insulin-secretion, we hypothesized that lower plasma levels of ghrelin, due to more extensive gastric resection, may play an important role in the early glycemic control after surgery in a first time as we have showed in a recent report [18].

This study presents several limitations as the length of follow-up that was too short(36 months) and no measurement of the volume of the residual stomach was performed. Since plain techniques to determine exactly the size of gastric sleeve cannot be found in literature [4,13], we assumed -as suggested by some authors [14]- that the standardization of the technique performed by only one surgeon could have given similar results about the sleeve size.

## 5 Conclusion

In conclusion our results suggest that poorly DM2 control after LSG are independent from HRGV. The removal of a larger gastric volume was only associated with a significant reduction in %HbA1c during the first year after surgery showing a probably implication in the regulation of the glucose metabolism in this timing. Further studies with a wider cohort of patients and a longer follow-up are needed in order to assess the effects of HRGV on gut hormonal changes responsible for DM2 control after LSG.

## Ethical approval

Not applicable.

## Funding

All the authors declare that they have no source of funding.

## Author contributions

Clementi M, Carandina S and Sista F contributed the original idea and steasure of the manuscript.

Clementi M, Abruzzese V and Carandina S contributed by conceptualization and performing the surgical procedures.

Sista F, Abruzzese V and Guadagni S contributed by collecting all the data.

Guadagni S. and Sista F contributed by conceptualization and revision of the manuscript.

The final manuscript has been read and approved by all named authors and that there are no other persons who satisfied the criteria for authorship but are not listed. We further confirm that the order of authors listed in the manuscript has been approved by all of us.

## Conflicts of interest

All the authors declare that they have no conflict of interest.

## Research registration number

Researchregistry4289.

## Guarantor

Federico Sista, PhD.

## Financial support and declarations of interest

The authors report no proprietary or commercial interest in any product mentioned or concept discussed in this article.

## Consent

There is no need for ethical approval because it is an observational study. Written informed consent was obtained from the patients for publication of this observational study.

## Provenance and peer review

Not commissioned externally peer reviewed.
